# Bacterial Prevalence in Skin, Urine, Diarrheal Stool, and Respiratory Samples from Dogs

**DOI:** 10.3390/microorganisms10081668

**Published:** 2022-08-19

**Authors:** Dong-Chan Moon, Ji-Hyun Choi, Naila Boby, Hee-Young Kang, Su-Jeong Kim, Hyun-Ju Song, Ho-Sung Park, Min-Chan Gil, Soon-Seek Yoon, Suk-Kyung Lim

**Affiliations:** 1 Bacterial Disease Division, Animal and Plant Quarantine Agency, 177 Hyeksin 8-ro, Gimcheon-si 39660, Korea; 2 Centre for Infectious Diseases Research, Korea Centers for Disease Control and Prevention, Division of Antimicrobial Resistance, Cheongju 28159, Korea; 3 Department of Microbiology and Medical Science, Chungnam National University School of Medicine, Daejeon 35015, Korea

**Keywords:** nationwide, companion animals, infections, bacteria

## Abstract

The emergence of bacterial infections in companion animals is a growing concern as humans can also be infected through the transmission of pathogenic bacteria. Because there have been few studies conducted on companion animals, the extent and significance of prevalence in veterinary practices remain unknown. This is the first nationwide surveillance report aimed at elucidating the prevalence pattern and associated infections of isolated bacteria from dogs in Korea. Bacterial isolates were collected from seven different laboratories participating in the Korean Veterinary Antimicrobial Resistance Monitoring System from 2018 to 2019. The samples were obtained from the diarrheal stool, skin/ear, urine, and respiratory samples of veterinary hospital-visited dogs. Isolation and identification of bacterial species was carried out using a bacterial culture approach and then confirmed with matrix-assisted laser desorption/ionization-time-of-flight mass spectrometry (MALDI-TOF) and polymerase chain reaction (PCR). Out of 3135 isolates in dogs, 1085, 1761, 171, and 118 were extracted from diarrheal stool, skin/ear, urine, and respiratory samples, respectively. The overall prevalence of bacteria was higher among two age groups (1–5 and 6–10 years) with a 66.5 percent prevalence. This study showed that *Escherichia coli* was the most prevalent species among isolated bacterial species of diarrheal and urine origin, whereas *Staphylococcus pseudintermedius* was the most prevalent among skin and respiratory sample isolates. The data on the prevalence of bacteria for each dog specimen could provide basic information to estimate the extent of bacterial infection and antimicrobial resistance development and to guide veterinarians in therapeutic decisions in clinical practices throughout Korea.

## 1. Introduction

The dog is one of the most popular companion animals that is beginning to play an important role as a member of the family not only in Korea, but also around the world. Recent practices in veterinary clinics have a drastic impact on these animals [1]. The population of dogs housed as companion animals or in animal shelters is at increased risk of carrying and spreading a variety of pathogens to both animals and humans. Some of the common pathogens of dogs are also important public health pathogens. Moreover, the increase in morbidity and mortality in companion animals due to bacterial infections is also a growing concern around the globe [2]. Some factors that contribute to the introduction, persistence, and spread of pathogens include lack of proper veterinary care, high animal population densities, limited funding, stressful and unsanitary housing conditions, adaptation across state boundaries, high animal turnover, and an increase in the pet ownership rate [3]. Companion animals might be an integral part of the bacterial infection transfer due to their close and direct contact with humans [4].

The distribution of bacterial population has been described previously for several geographical regions around the world including Europe [5], South America [6], North America [7,8], Australia [9,10], and Asia [11,12,13]. However, although there are diverse similarities between these regional investigations, there are notable differences in bacterial prevalence across the world and differences within separate geographic regions of even a single country depicted that a more targeted evaluation of local or regional bacterial prevalence is needed [7,8]. Moreover, most studies in different countries investigated trends or patterns in prevalence by focusing on a specific pathogenic bacterium (e.g., *Salmonella*) or a specific organ/system (e.g., urinary tract infections) and their susceptibility [4,14,15,16] in dogs. However, only a few reports from veterinary diagnostic laboratories have provided information on trends and patterns in bacterial isolate prevalence from clinical samples of dogs that were submitted over the course of 10–20 years [17,18].

Unfortunately, researchers have followed the same trend in Korea. Bacterial studies are not limited to susceptibility and resistance patterns, but regional bacterial prevalence surveillance in dogs is also scarce [4,16,19,20]. As bacterial infections are a constantly evolving situation, studies involving regular monitoring of bacterial prevalence are needed in order to develop up-to-date control strategies and the rational selection of treatment strategies. Thus, this study aimed to report the nationwide prevalence of pathogenic bacteria in dog specimens in all metropolitan cities of Korea.

## 2. Materials and Methods

**Figure 1:**
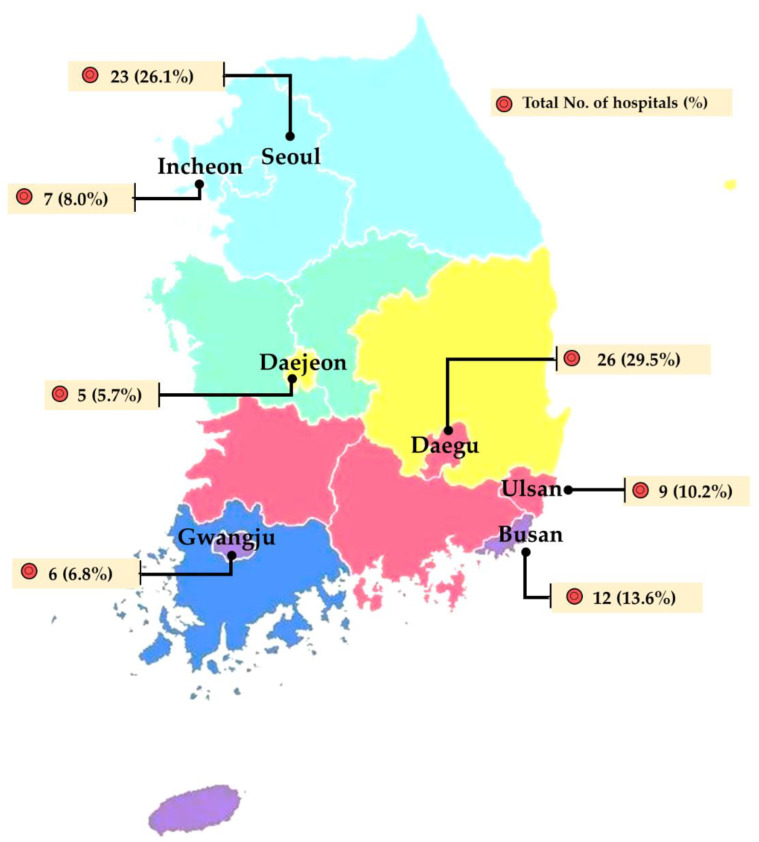
Demographic presentation and the total number of hospitals (%) that participated in this study from each city. In the figure, the hospitals that appeared more than one time in the study period counted as one while calculating the total number of hospitals.

In the presented report, bacterial isolates were collected from 7 different laboratories participating in the Korean Veterinary Antimicrobial Resistance Monitoring System from 2018 to 2019. The isolates were obtained from the diarrheal stool, skin/ear, urine, and respiratory samples of sick dogs. Samples were placed on ice and transported to the laboratories/centers participating in the monitoring system within 6 h of their collection. However, the isolates were collected in proportion to the number of veterinary hospitals in each city (Figure 1).

For the bacterial isolation, we followed a similar protocol as described in our previous report [21]. Briefly, the sampled swabs were directly plated on a culture plate, i.e., 5% defibrinated sheep blood agar (Hangang, Gunpo, Korea), and MacConkey agar plates (MAC, BD, Spark, Baltimore, MD, USA), and incubated overnight at 37 °C for 24 h under aerobic and anaerobic conditions, separately. In order to exclude the contaminant samples, only 1–2 major colonies showing morphological differences in one culture plate were selected for further analysis.

Matrix-assisted laser desorption/ionization–time-of-flight mass spectrometry (Microflex LT/SH spectrometer, Bruker, Bruker Daltonics, Bremen, Germany) was applied for the identification of each colony. However, *Staphylococcal* species were identified by polymerase chain reaction using a previously reported method of identification [22].

Finally, collected data were analyzed using Excel (Microsoft Office, Redmond, WA, USA) and GraphPad Prism 7 software (San Diego, CA, USA). The statistical methodology included Student’s t-test, and all comparisons were two-tailed. Categorical variables were expressed as numbers and percentages. The threshold for statistical significance was set to a *p*-value of less than 0.05.

## 3. Results

### 3.1. Prevalence Pattern among Different Age Groups

A total of 3135 bacteria were isolated from diarrheal stool, skin, urine, and respiratory specimens during a nationwide surveillance study of veterinary hospital-visited sick dogs. As compared to other samples, bacterial isolation was more frequent in diarrheal stool and skin samples, accounting for 90.8% of all recoveries. When comparing prevalence among different age groups, the overall prevalence of bacteria was higher among two age groups (1–5 and 6–10 years) with a prevalence of 66.5%. Of note, bacteria recovered from respiratory samples showed a similar proportion among all age groups of dogs (Table 1).

### 3.2. Prevalence of Bacteria in Diarrheal Stool Samples

A total of 1085 isolates from 44 different bacterial species were identified from the dogs’ diarrheal stool samples. About 92.8% belonged to 11 different bacterial species. Among all the identified bacteria, *E. coli* accounted for 56.4%, followed by *Clostridium perfringens* (*C. perfringens*) (10.7%), *Klebsiella pneumoniae* (*K. pneumoniae*), *Proteus mirabilis* (*P. mirabilis*), and *Enterococcus faecalis* (*E. faecalis*) (5.3%). *Escherichia coli (E. coli)* was the most predominant bacteria in all provinces; however, the prevalence of other bacteria was different from one city to another (Table 2).

**Figure 2:**
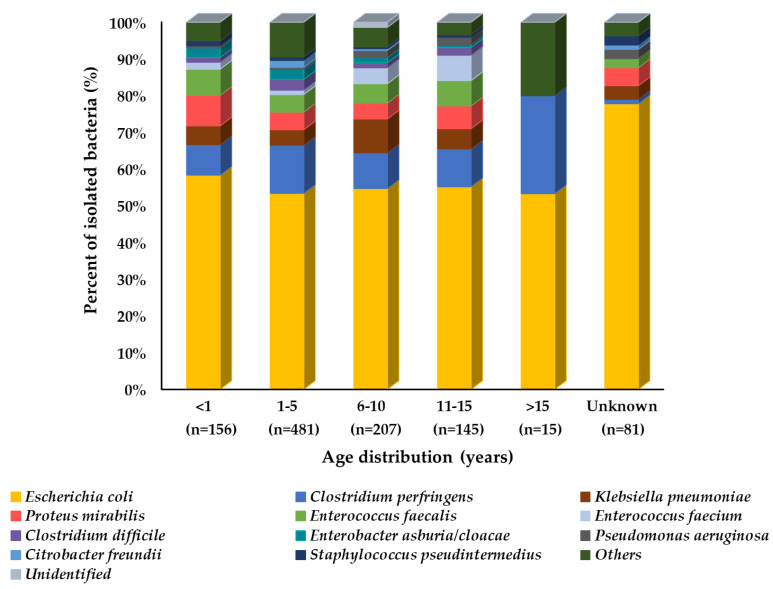
Prevalence of isolated bacteria (%) from dogs’ diarrheal stool by age group (n = 1085). In the graph, “n” represents the number of recovered isolates.

When comparing prevalence among identified isolates from all age groups of dogs, the overall distribution pattern was similar, as *E. coli* was most frequently observed in all age groups. However, some bacterial species may differ in their prevalence among different age groups, such as *P. mirabilus* and *C. perfringens*, which were most prevalent in the <1- and >15-year-old age groups of dogs, respectively, as compared to other age groups, as shown in Figure 2.

### 3.3. Prevalence of Bacteria in Skin Samples

A total of 1761 isolates belonging to 75 species were identified in the dogs’ skin samples. Among the isolated bacterial species, the *Staphylococcus pseudintermedius* (*S. pseudintermdeius*) (49.1%) was most prevalent, followed by *Staphylococcus schleiferi* (*S. schleiferi*) (17.7%). About eight bacterial species had a relatively low prevalence range of 1–>7%. The prevalence of bacterial species was similar in all cities with *S. pseudintermedius* being the highest (Table 3).

**Figure 3:**
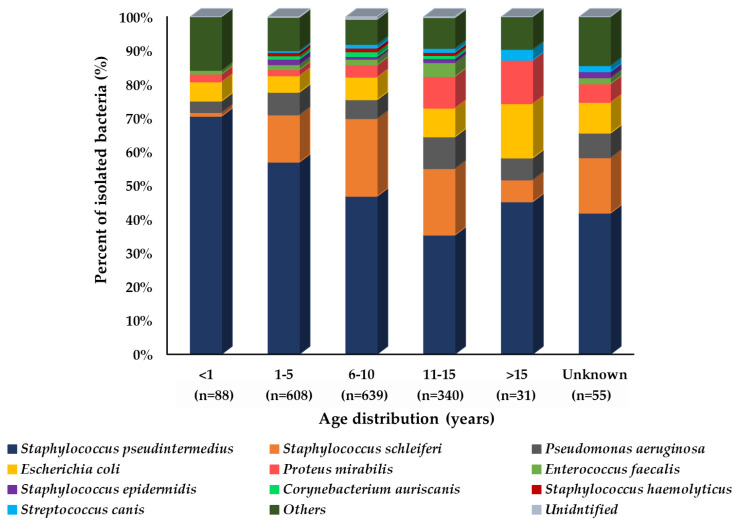
Prevalence of isolated bacteria (%) from dogs’ skin specimens by age group (n = 1761). In the graph, “n” represents the number of recovered isolates.

A comparison of isolated bacterial prevalence by age showed that for each bacterial species, the distribution pattern varied among different groups. *S. pseudintermedius* was isolated in a much higher proportion in younger age groups (<1–5 years of age). It especially had a much higher prevalence of 70.5% in the less than 1-year-old age group of dogs as compared to others (35.3–56.9%). *S. schleiferi* was the second most prevalent bacteria in the middle age group as compared to the younger and old age groups, as shown in Figure 3.

### 3.4. Prevalence of Bacteria in Urine Samples

A total of 171 isolates were identified from urine samples of dogs during nationwide surveillance studies. Among the isolated bacteria, *E. coli* was the most prevalent species of bacteria with a prevalence of 29.7%, followed by *P. mirabilus* (20.3%) and *S. pseudintermedius* (15.1%). The overall prevalence for these three species was 65.1%, whereas other species had a prevalence range of 1.7 to 7.6%. The identified bacterial species followed a similar pattern of prevalence for all cities except Busan and Daegu. *S. canis* and *E. faecalis* were the most prevalent bacteria in Busan and Daegu, respectively. However, the prevalence of identified bacterial species was different from one city to another (Table 4).

**Figure 4:**
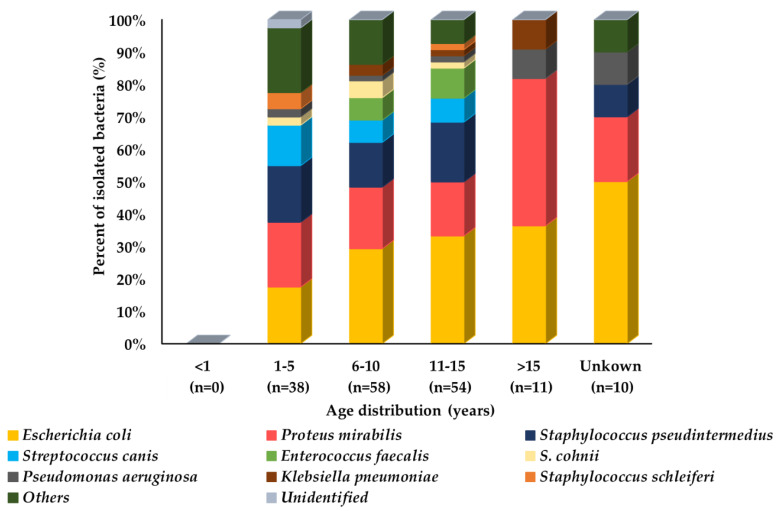
Prevalence of isolated bacteria (%) from dogs’ urine specimens by age group (n = 171). In the graph, “n” represents the number of recovered isolates.

Among all age groups, different bacterial species have different prevalence patterns. *E. coli* and *P. mirabilia* prevalence increased with an increase in age. However, *S. pseudintermedius* showed a similar prevalence among all age groups except the old age group of dogs, whereas other bacterial species with <10% prevalence were isolated mainly from the middle age group of dogs. Moreover, *K. pneumoniae* and *P. aeruginosa* were more frequently isolated in the old age group of dogs as compared to other age groups. *S. canis* was more prevalent in younger age groups and *E. faecalis* was more prevalent in middle and older age groups of dogs (Figure 4).

### 3.5. Prevalence of Bacteria from Respiratory Tract

From the respiratory tract samples of dogs, overall, 118 isolates were identified. Although the number of isolates was small compared with the other samples, a variety of bacterial species (40 species) was isolated from respiratory samples. Like the isolates from the skin, the maximum isolated bacteria belonged to the *S. pseudintermedius* species with a prevalence of 33.3%. However, the prevalence of other bacterial species was relatively lower in this case as compared to the other samples collected from the dogs. Moreover, *Rothia nasimurium (R. nasimurium)* (3.3%), *Enterobacter aerogenes* (*E. aerogenes*) (2.5%), and *Pasteurella canis* (*P. canis*) (2.5%) were bacteria only isolated from the respiratory samples (Table 5).

**Figure 5:**
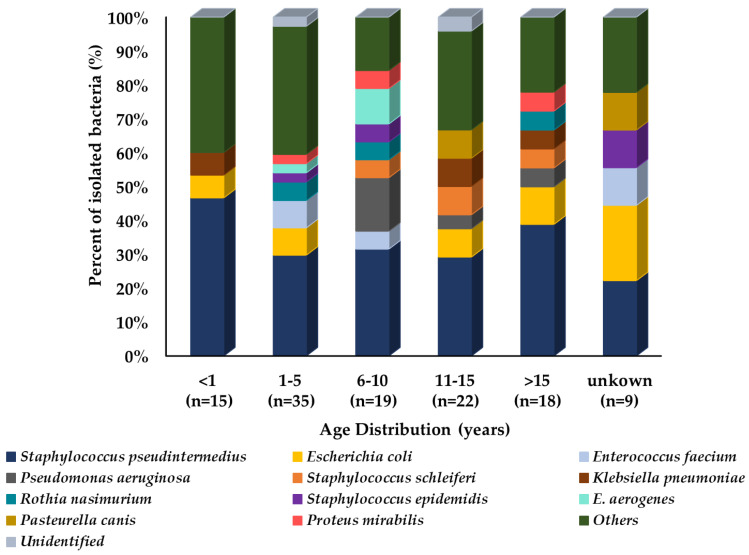
Prevalence of isolated bacteria (%) from dogs’ respiratory specimens by age group (n = 118). In the graph, “n” represents the number of recovered isolates.

When comparing prevalence with age groups, the isolated bacterial species showed differences in their prevalence patterns among age groups. *E. coli* showed a similar prevalence among all age groups except the 6–10-year-old age group of dogs. *P. mirabilus* was more prevalent in middle and older age groups, but less prevalent in the younger age group of dogs. *E. faecium* showed more prevalence in middle age groups as compared to other age groups of dogs. Moreover, *K. pneumoniae* was most prevalent in the older age group followed by the younger age group, and the middle age group had the least prevalence. However, *S. pseudintermedius* showed almost equal prevalence among all age groups (Figure 5).

## 4. Discussion

In this study, we reported the nationwide bacterial prevalence in dog specimens for the first time in Korea. We identified the pathogenic bacteria associated with different age groups of dogs to illuminate the age-dependent prevalence transitions. Among our collected isolates from major sites of infection, the most prevalent bacterial species in dogs was *E. coli* followed by *S. pseudintermedius*, which agrees with previous reports by Han et al., Moon et al., and EM et al. from Korea and Belgium. However, the distribution of bacterial species was dependent on the sampling hosts [23,24,25].

We presented the bacterial prevalence in different age groups of dogs. Dogs from 1 to 10 years of age are more vulnerable to bacterial infections with an increased prevalence compared to the other age groups. As there are only limited reports found in the literature that previously regarded bacterial prevalence and age association, it is difficult to compare the presented findings with other reports. In the collected diarrheal stool samples from dogs, the *E. coli* strains were not associated with a specific age group (*p* > 0.05) and equally affected all age groups with diarrhea (*p* ≤ 0.05).

These findings are consistent with the study from the West Indies that reported an insignificant association of *E. coli* prevalence with age in the case of the diarrheic dogs. In a similar study, Adesiyun et al. reported two more bacterial species as more prevalent in diarrheic dogs, which is contrary to our findings [26]. In addition, since gastrointestinal disorders were also caused by various factors such as microbial dysbiosis or metabolomic changes; thus, a comprehensive analysis of diarrhea samples is needed [27].

*S. pseudintermedius* is part of the commensal biota and is an etiological agent for skin infections, especially pyoderma and otitis [28]. Our results showed that the most frequently isolated *staphylococcal* species among all samples were from the skin and urine samples, which is also consistent with the results of other studies [29,30]. Similarly, in the findings of presented bacterial prevalence, we identified the *S. canis* bacteria in the skin and urine samples of dogs. Our findings are in similar to those in the literature that reported *S. canis* as a beta-hemolytic Lancefield group G *streptococcus* that colonizes the skin and the reproductive tract of dogs [31,32,33]. This zoonotic pathogen is known to be associated with important genitourinary tract infections, septicemia, and skin infections in dogs [32,34,35]. However, *Enterococcus* spp. was isolated from all the samples of dogs at a lower frequency.

Studies from the American Animal Hospital Association [36] and College of Veterinary Medicine, North Carolina State University [37] reported that *E. coli*, *Proteus* spp., *Klebsiella* spp., *Pseudomonas* spp., and *Enterococcus* spp. were the six most prevalent bacterial species in the urine of dogs with urinary tract infections (UTIs). These findings are consistent with our analysis of the dogs’ urine specimens.

This report was consistent with some other international reports. In line with the ComPath project of Europe [38], our findings showed that *S. pseudintermedius* is the major isolate from the skin samples, followed by *E. coli* and *P. aeruginosa*.

Moreover, our results showed that among the four types of samples, *E. coli* was the most prevalent isolate in diarrheal stool and urine samples and the third most prevalent in the skin samples of dogs; however, no isolate was extracted from the respiratory tract. These findings were consistent with the previous literature [16,25,39]. As Hammerum and Heuer reported, *E. coli* is classified into various pathotypes due to its role in intestinal and extra-intestinal infections such as gastroenteritis, skin and soft tissue infections (SSTI), and urinary tract infections (UTIs) in humans and animals [40]. This showed that the chance of *E. coli* infection is more likely in the skin, and gastrointestinal and urinary tract than in other sites. On the other hand, *P. mirabilus* was identified in all the samples with a relatively higher prevalence in urine and diarrheal stool samples as compared to other samples. These findings are consistent with many other reports about the pathogenic, commensal, or transient association of *P. mirabilus* with multiple sites including the nose, ear, eye, respiratory tract, burns, wounds, and throat [41,42]. As O’Hara et al. and Jacobsen et al. reported in their studies, the majority of *P. mirabilus* are associated with urinary tract infections (UTIs), but in some cases, similar *P. mirabilus* strains were also present in the gastrointestinal tract together with UTIs [41,43].

Among the isolates collected from the respiratory tract samples of dogs, *S. pseudintermedius* was the most prevalent bacteria followed by *E*. *coli*, *E. faecium*, *K. pneumoniae*, *R. nasimurium, P. canis,* and *P. mirabilis*. Our results were different from other studies. *Pasteurella* spp. and *B. bronchiseptica* were the most predominant species in South Africa, the UK, the US, and Japan [38,44,45,46]. However, these bacteria species were rarely detected in our study. *S. pseudintermedius* is a major opportunistic pathogen that is involved in numerous canine infections including respiratory tract infections. It is known to be associated with the colonization of the skin and mucous membrane of companion animals [47,48,49]. Although *R. nasimurium* and *P. canis* were rarely detected in our study, these bacteria are known to cause respiratory tract infections in dogs. *P. canis* is especially known to cause zoonotic infections, mainly transmitted through dog bites [50,51].

## 5. Conclusions

In this study, we explored the major bacterial species from four different infection sites of dogs. *E. coli* and *S. pseudintermedius* were major pathogenic bacteria of diarrheal stool, skin, and respiratory origin in Korea, whereas *E. coli* and *P. mirabilis* were predominantly present in the urine of dogs throughout Korea. These findings could provide important baseline measurements for upcoming bacterial dominance and associated infections in dogs. However, this study has some limitations, including the lack of provided information regarding the number of samples by region and season, sex, and age of the targeted animals, the severity of the condition of the animal, and, due to the lack of specific culture media and growth conditions, several human-relevant bacteria were not recovered. Furthermore, there is a need to study the associated infections and susceptibility profile of these bacteria to provide detailed information for the prevention and treatment of bacterial diseases in the future. Bacterial prevalence studies are not only important to attenuate the overuse of antimicrobials in veterinary practices, but also help to reduce the spread of bacterial infections among companion animals and humans. This report could decipher the sufficient information on the selection of antimicrobials and development of vaccines for the treatment and prevention of infections in dogs.

## Figures and Tables

**Table 1:** Number of recovered isolates from four targeted samples and different age groups of dogs.

Sampling Site	No. of Isolates (%)						Total
	<1 year	1–5 years	6–10 years	11–15 years	>15 years	Unknown	
**Diarrhea**	156 (14.4)	481 (44.3)	207 (19.1)	145 (13.4)	15 (1.4)	81 (7.5)	1085 (34.6)
**Skin**	88 (5.0)	608 (34.5)	639 (36.3)	340 (19.3)	31 (1.8)	55 (3.1)	1761 (56.2)
**Urine**	-	38 (22.2)	58 (33.9)	54 (31.6)	11 (6.4)	10 (5.8)	171 (5.5)
**Respiratory**	15 (12.7)	35 (29.7)	19 (16.1)	22 (18.6)	18 (15.3)	9 (7.6)	118 (3.8)
**Total**	259 (8.3)	1162 (37.1)	923 (29.4)	561 (17.9)	75 (2.4)	155 (4.9)	3135 (100)

**Table 2:** Recovered isolate prevalence in diarrheal stool of dogs during nationwide surveillance (2018–2019).

Bacterial Species	No. of Isolates (%)							
	Seoul (*n* = 502)	Busan (*n* = 123)	Daegu (*n* = 29)	Incheon (*n* = 55)	Gwangju (*n* = 41)	Daejeon (*n* = 258)	Ulsan (*n* = 77)	Total (*n* = 1085)
*E. coli*	227 (45.2)	116 (94.3)	26 (89.7)	30 (54.5)	36 (87.8)	145 (56.2)	32 (41.6)	612 (56.4)
*C. perfringens*	63 (12.5)	1 (0.8)	1 (3.4)	-	-	37 (14.3)	14 (18.2)	116 (10.7)
*K. pneumoniae*	36 (7.2)	2 (1.6)	-	6 (10.9)	1 (2.4)	8 (3.1)	5 (6.5)	58 (5.3)
*P. mirabilis*	22 (4.4)	-	-	5 (9.1)	-	23 (8.9)	8 (10.4)	58 (5.3)
*E. faecalis*	31 (6.2)	-	-	1 (1.8)	1 (2.4)	21 (8.1)	3 (3.9)	57 (5.3)
*E. faecium*	20 (4.0)	-	-	-	-	6 (2.3)	2 (2.6)	28 (2.6)
*C. difficile*	22 (4.4)	-	-	-	-	-	-	22 (2.0)
*E. asburia/cloacae*	15 (3.0)	-	-	-	-	2 (0.8)	4 (5.2)	21 (1.9)
*P. aeruginosa*	5 (1.0)	1 (0.8)	-	7 (12.7)	-	-	-	13 (1.2)
*C. freundii*	9 (1.8)	-	-	1 (1.8)	1 (2.4)	-	-	11 (1.0)
*S. pseudintermedius*	8 (1.6)	-	-	-	2 (4.9)	1 (0.4)	-	11 (1.0)
Others *	41 (8.2)	3 (2.4)	2(6.9)	5 (9.1)	-	15 (5.8)	9 (11.7)	75 (6.9)
Unidentified	3 (0.6)	-	-	-	-	-	-	3 (0.3)

* Others: 33 species.

**Table 3:** Recovered isolate prevalence in skin samples of dogs during nationwide surveillance (2018–2019).

Bacterial Species	No. of Isolates (%)							
	Seoul (*n* = 375)	Busan (*n* = 359)	Daegu (*n* = 185)	Incheon (*n* = 141)	Gwangju (*n* = 100)	Daejeon (*n* = 457)	Ulsan (*n* = 144)	Total (*n* = 1761)
*S. pseudintermedius*	170 (45.3)	166 (46.2)	93 (50.3)	40 (28.4)	67 (67.0)	252 (55.1)	76 (52.8)	864 (49.1)
*S. schleiferi*	78 (20.8)	68 (18.9)	22 (11.9)	21 (14.9)	23 (23.0)	70 (15.3)	29 (20.1)	311 (17.7)
*P. aeruginosa*	20 (5.3)	23 (6.4)	25 (13.5)	20 (14.2)	2 (2.0)	22 (4.8)	6 (4.2)	118 (6.7)
*E. coli*	21 (5.6)	21 (5.8)	20 (10.8)	12 (8.5)	3 (3.0)	28 (6.1)	12 (8.3)	117 (6.6)
*P. mirabilis*	14 (3.7)	18 (5.0)	1 (0.5)	5 (3.5)	-	34 (7.4)	3 (2.1)	75 (4.3)
*E. faecalis*	5 (1.3)	1 (0.3)	-	24 (17.0)	-	5 (1.1)	1 (0.7)	36 (2.0)
*S. epidermidis*	6 (1.6)	5 (1.4)	3 (1.6)	-	-	4 (0.9)	2 (1.4)	20 (1.1)
*C. auriscanis*	14 (3.7)	1 (0.3)	-	-	-	1 (0.2)	2 (1.4)	18 (1.0)
*S. haemolyticus*	1 (0.3)	5 (1.4)	1 (0.5)	1 (0.7)	1 (1.0)	5 (1.1)	2 (1.4)	16 (0.9)
*S. canis*	5 (1.3)	6 (1.7)	3 (1.6)	1 (0.7)	-	1 (0.2)	-	16 (0.9)
Others *	39 (10.4)	44 (12.3)	15 (8.1)	17 (12.1)	4 (4.0)	33 (7.2)	11 (7.6)	163 (9.3)
Unidentified	2 (0.5)	1 (0.3)	2 (1.1)	-	-	2 (0.4)	-	7 (0.4)

* Others: 65 species.

**Table 4:** Recovered isolate prevalence in urine samples of dogs during nationwide surveillance (2018–2019).

Bacterial Species	No. of Isolates (%)							
	Seoul (*n* = 35)	Busan (*n* = 51)	Daegu (*n* = 16)	Incheon (*n* = 18)	Gwangju (*n* = 3)	Daejeon (*n* = 38)	Ulsan (*n* = 11)	Total (*n* = 171)
*E. coli*	16 (46.0)	6 (11.8)	3 (18.8)	4 (22.2)	2 (66.7)	15 (39.5)	5 (45.5)	51 (29.7)
*P. mirabilis*	7 (20.0)	4 (7.8)	2 (12.5)	12 (66.7)	-	10 (26.3)	-	35 (20.3)
*S. pseudintermedius*	2 (5.7)	10 (19.6)	3 (18.8)	1 (5.6)	-	10 (26.3)	-	26 (15.1)
*S. canis*	-	13 (25.5)	-	-	-	-	-	13 (7.6)
*E. faecalis*	1 (2.9)	1 (2.0)	5 (31.3)	-	-	-	2 (18.2)	9 (5.2)
*S. cohnii*	2 (5.7)	3 (5.9)	-	-	-	-	-	5 (2.9)
*P. aeruginosa*	-	2 (3.9)	1 (6.3)	-	1 (33.3)	-	1 (9.1)	5 (2.9)
*K. pneumoniae*	3 (8.6)	1 (2.0)	-	-	-	-	-	4 (2.3)
*S. schleiferi*	-	2 (3.9)	1 (6.3)	-	-	-	-	3 (1.7)
Others *	4 (11.4)	9 (17.6)	1 (6.3)	1 (5.6)	-	3 (7.9)	3 (27.3)	21 (12.2)
Unidentified	-	-	-	-	-	1 (2.6)	-	1 (0.6)

* Others: 13 species.

**Table 5:** Recovered isolate prevalence in respiratory samples of dogs during nationwide surveillance (2018–2019).

Bacterial Species	No. of Isolates (%)							
	Seoul (*n* = 45)	Busan (*n* = 7)	Daegu (*n* = 13)	Incheon (*n* = 38)	Gwangju (*n* = 7)	Daejeon (*n* = 4)	Ulsan (*n* = 6)	Total (*n* = 118)
*S. pseudintermedius*	12 (26.7)	2 (28.6)	5 (38.5)	20 (52.6)	1 (14.3)	-	-	40 (33.3)
*E. coli*	2 (4.4)	1 (14.3)	3 (23.1)	-	2 (28.6)	1 (25.0)	1 (16.7)	10 (8.3)
*E. faecium*	3 (6.7)	-	-	1 (2.6)	1 (14.3)	-	-	5 (4.2)
*P. aeruginosa*	1 (2.2)	-	1 (7.7)	3 (7.9)	-	-	-	5 (4.2)
*S. schleiferi*	1 (2.2)	1 (14.3)	-	1 (2.6)	-	-	1 (16.7)	4 (3.3)
*K. pneumoniae*	3 (6.7)	-	-	-	-	1 (25.0)	-	4 (3.3)
*R. nasimurium*	4 (8.9)	-	-	-	-	-	-	4 (3.3)
*S. epidermidis*	2 (4.4)	-	1 (7.7)	-	-	-	-	3 (2.5)
*E. aerogenes*	-	-	-	3 (7.9)	-	-	-	3 (2.5)
*P. canis*	2 (4.4)	-	-	-	1 (14.3)	-	-	3 (2.5)
*P. mirabilis*	1 (2.2)	-	1 (7.7)	1 (2.6)	-	-	-	3 (2.5)
Others *	14 (31.1)	3 (42.9)	2 (15.4)	9 (23.7)	2 (28.6)	2 (50.0)	4 (66.7)	36 (30.0)
Unidentified	-	-	-	1 (2.6)	-	-	1 (16.7)	2 (1.7)

* Others: 29 species.

## Data Availability

The data that support the findings of this study are available from the corresponding author upon reasonable request.
